# Ultrasound microbubble-mediated RNA interference targeting WNT1 inducible signaling pathway protein 1(WISP1) suppresses the proliferation and metastasis of breast cancer cells

**DOI:** 10.1080/21655979.2022.2068738

**Published:** 2022-04-28

**Authors:** Faying Fang, Weizhi Xu, Jian Zhang, Jin Gu, Gaoyi Yang

**Affiliations:** a Department of Special Examination, Maternal and Child Health Hospital of Chun’an County, Hangzhou, Zhejiang, China; b Department of Ultrasound, Sanmen People’s Hospital, Taizhou, Zhejiang, China; c Department of Ultrasound, Pingyi County Hospital of Traditional Chinese Medicine, Linyi, Shandong, China; d Department of Ultrasound, Chongqing Public Health Medical Center, Chongqing, Shandong, China

**Keywords:** Ultrasound irradiation, SonoVue microbubble, RNA interference, WNT1 inducible signaling pathway protein 1

## Abstract

In the context of relatively sufficient research that annotated WNT1 inducible signaling pathway protein 1 (WISP1) as a promoting factor in tumor progression of breast cancer, and identified the effects of ultrasound microbubble technology on enhancing the transfection efficiency and achieving better gene interference, this study managed to investigate the effects of ultrasound microbubble-mediated siWISP1 transfection on proliferation and metastasis of breast cancer cells. To achieve our research objectives, the expression of WISP1 in breast cancer tissues was retrieved from GEPIA website, and the viability of breast cancer cells (SK-BR-3 and MCF7) was assessed by 3-(4,5-dimethylthiazol-2-yl)-2,5-diphenyltetrazolium bromide (MTT) assay for ultrasound intensity screening. After the transfection of siWISP1 by ultrasound microbubble or lipofectamine 6000, the content of WISP1 secreted by cells was detected through Enzyme-linked immunosorbent assay (ELISA), and WISP1 expression in cells was determined by quantitative reverse transcription polymerase-chain reaction (qRT-PCR). Besides, the cell invasion, migration, and proliferation were evaluated by wound healing, transwell, and EdU assays, respectively. In accordance with experimental results, WISP1 was highly expressed in breast cancer tissues, and the 1 W/cm^2^ intensity was the onset of a notable decrease in cell viability. Compared with lipofectamine 6000 transfection, the transfection of siWISP1 mediated by ultrasound microbubble further reduced the expression of WISP1, and meanwhile suppressed cell invasion, migration, and proliferation. Collectively, ultrasound microbubble-mediated transfection of siWISP1 worked rather effectively in improving transfection efficiency and inhibiting the progression of breast cancer.

## Graphical abstract

**Figure:**
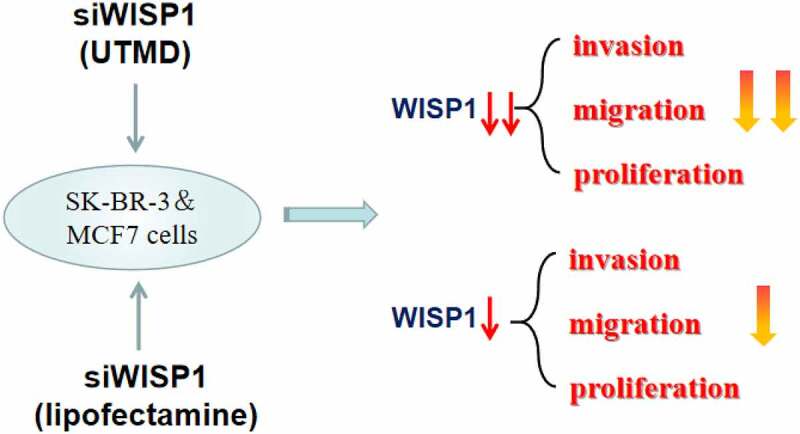

## Highlights

- WISP1 expression was elevated in breast cancer tissues.
- Lipofectamine 6000-mediated transfection of siWISP1 suppressed the invasion, migration and proliferation of breast cancer cells.
- Ultrasound microbubble enhanced the transfection efficiency and promoted the antitumor activity of siWISP1.
- Ultrasound microbubble can be a new delivery vector for siWISP1 and a novel treatment for breast cancer.

## Introduction

Breast cancer is a commonly diagnosed malignant tumor in women with an increasing incidence, seriously endangering women’s life and health [1]. With the continuous development of molecular biology and genomics, progress has been made in the treatment of breast cancer, and gene therapy becomes a hotspot in treating this disease [2]. The selection of gene delivery vectors largely affects the efficacy of gene therapy. Although viral vectors have high transfection efficiency, such vectors have limited clinical application due to the disadvantages, such as potential toxicity, immune response, mutagenicity, and carcinogenicity, whereas non-viral vectors often have lower transfection efficiency than viral vectors [3]. Therefore, how to prepare safe and efficient gene delivery vectors becomes a new direction in gene therapy research for breast cancer.

Recently, ultrasound microbubble contrast agents have gained much attention as a novel non-viral vector for their promising application in tumor gene therapy by dint of simple preparation, high gene transfection efficiency, and no immunogenicity [4]. The successful application of ultrasound microbubbles to gene therapy mainly depends on ultrasound targeted microbubble destruction (UTMD) technology [5]. The mechanism of UTMD is to break the microbubbles carrying exogenous genes in the target tissues or organs by ultrasonic irradiation, so as to release the exogenous gene. At the same time, the cavitation effect caused by microbubble explosion can increase the cell membrane permeability and promote the gene to enter the cells for transfection [6]. Since UTMD has shown great potential in breast cancer treatment as a novel transfection modality [7,8], it is expected to become a safe, efficient, and targeted method for gene therapy of breast cancer. Besides, RNA interference (RNAi) therapy is one of the common methods for gene therapy of breast cancer mediated by ultrasound microbubble contrast agents [9]. RNAi technology, a post-transcriptional gene silencing phenomenon triggered by double stranded RNAs with homology, can cause degradation of the mRNA, thereby inhibiting the function of the gene with high specificity and effectiveness [10]. However, RNAi technology has a limited application owing to the poor stability and low cellular uptake rate of siRNA, which combined with microbubbles under ultrasound irradiation can improve the safety of double stranded RNA transport *in vivo* and the cellular uptake rate, thereby enhancing gene therapy efficacy [9]. For example, Zhao et al. prepared ultrasound microbubbles with FOXA1-siRNA and porphyrin, and unveiled that low-frequency ultrasound together with microbubbles could promote porphyrin uptake and siRNA transfection, generating positive therapeutic effects on breast cancer [11]. Therefore, UTMD-mediated RNAi may become a therapeutic option for breast cancer, but the involvement of various genes mediated by UTMD-inducible RNAi in breast cancer needs to be further explored.

As a part of breast cancer gene signal, WNT1 inducible signaling pathway protein 1 (WISP1) is closely related to the proliferation activity of breast cancer cells, which belongs to the CCN protein family and is a downstream target protein of the Wnt/β-catenin pathway [12,13]. WISP1 is expressed in human multiple organs, functions in regulating cell proliferation and metastasis, and plays an important role in inflammation, wound repair, angiogenesis, and tumorigenesis [14–16]. Based on the previous studies, WISP1 has been found to be high-expressed in a variety of tumors and is closely related to poor prognosis of tumor patients, including oral squamous cell carcinoma [17], esophageal squamous cell carcinoma [18], and pancreatic ductal adenocarcinoma [19], thus it can serves as a clinical marker for above cancers. In addition, it is worth noting that WISP1 is significantly increased in breast cancer patients with poor prognosis [20], and can promote the proliferation, invasion, and metastasis of breast cancer cells [21], which correlates with invasive breast cancer oncogenesis [22]. Accordingly, WISP1 may serve as a potential cancer promoting gene to unfold research into breast cancer.

On the above basis, we hypothesized that silencing WISP1 may suppress the biological characteristics of breast cancer cells, and that UTMD-mediated RNA interference may enhance the effects of silenced WISP1 on breast cancer cells. Thus, this study aimed to investigate the effects of ultrasound microbubble technology on RNAi targeting WISP1 and on proliferation and metastasis of breast cancer cells. The goal of our study is to explore the possibility and feasibility of UTMD-delivered silenced WISP1 in the gene therapy of breast cancer.

## Materials and methods

### Cell culture and treatment

Breast cancer cell lines (SK-BR-3 (HTB-30) and MCF7 (HTB-22)) and culture media were obtained from American Type Culture Collection (ATCC, USA). SK-BR-3 cells were cultivated in McCoy’s 5a Medium Modified (30–2007) supplemented with 10% fetal bovine serum (FBS, 164,210–500, Procell). MCF7 cells were cultured in Eagle’s Minimum Essential Medium (30–2003) added with 0.01 mg/ml human recombinant insulin (91077C, Sigma-Aldrich, USA) and 10% FBS. The incubation was carried out at 37°C with 5% CO_2_ (Heracell Vios 160i CR CO2 incubator, 51,033,770, Thermo Scientific, USA).

### Gene profiling analysis

Gene Expression Profiling Interactive Analysis (GEPIA) (http://gepia.cancer-pku.cn/) was used to analyze the expression of WISP1 in breast cancer (n = 1085) and normal samples (n = 291).

### Transfection

WISP1 small interference RNA (siWISP1, siG13723182938-1-5) and siRNA negative control (siNC, siN0000001-1-5) were obtained from Ribobio (China). Lipofectamine 6000 transfection reagent (C0526, Beyotime, China) was adopted to accomplish the transfection of siWISP1 or siNC into SK-BR-3 and MCF7 cells according to the manufacturer’s specification. In short, cells were inoculated into 6-well plates at a density of 3 × 10^5^ cells/well until 80% confluence was reached. Thereafter, Lipo6000 (5 μl) and 100 pmol of siWISP1 or siNC were separately diluted in 125 μl of RPMI-1640 medium (PM150110, Procell, China) without serum to form the Lipo6000/oligonucleotide mixtures, followed by cell incubation with 250 μl of mixtures for 24 hours (h) at room temperature.

In terms of another transfection method using ultrasonic irradiation and SonoVue microbubbles (US), SonoVue (Bracco, Italy), the phospholipid-encapsulated six fluorinated sulfur (SF6) with an average diameter of 2.5 μm was used as previously described [23]. Briefly, 5 ml normal saline and 50 μl siWISP1 or siNC were added into the SonoVue microbubble before use. After cell incubation, 50 μl SonoVue microbubbles were introduced into each well of 24-well culture plates (concentration: 10%) and were irradiated with the increasing ultrasonic irradiation intensity (0, 0.5, 0.75, 1, 1.25 W/cm^2^) for 45 seconds (s) under the ultrasonic irradiation apparatus (Sonic15C, Fysiomed, Belgium). Quantitative reverse transcription polymerase-chain reaction (qRT-PCR) was utilized to analyze the transfection efficiency.

### Experimental grouping

SK-BR-3 and MCF7 cells were distributed into five groups: control group (normally cultured cells), siRNA-NC group (siNC-transfected cells by Lipofectamine 6000 transfection reagent), siRNA-WISP1 group (siWISP1-transfected cells by Lipofectamine 6000 transfection reagent), siRNA-NC+US group (cell transfection of siNC by US), and siRNA-WISP1+ US group (cell transfection of siWISP1 by US).

### 3-(4,5-dimethylthiazol-2-yl)-2,5-diphenyltetrazolium bromide (MTT) assay

MTT assay (M1020, Solarbio, China) was performed to access cell viability. Breast cancer cells were seeded in 96-well plates at a density of 3 × 10^3^ per well, and stimulated by various intensities of ultrasonic irradiation. Following incubation, the medium solution was replaced and 10 μl MTT solution was added for another 4-h incubation with cells. After the supernatants were discarded, 110 μl Formazan solution was added to each well and oscillated for 10 minutes (min). The absorbance value at 490 nm was estimated by a microplate reader (E0228, Beyotime, China).

### Enzyme-linked immunosorbent assay (ELISA)

After the transfection, cells were centrifuged (10,000 rpm) at 4°C for 5 min. Cell supernatants were then obtained for the determination of WISP1, which was conducted by ELISA kit (ab155445, abcam, UK) as per the instructions of manufacturer. In brief, an antibody specific for Human WISP-1 was coated on a 96-well plate. After centrifugation, cell supernatant was added into the wells. Thereafter, the wells were washed with Wash Buffer (300 μl) and added with 100 μl of biotinylated anti-Human WISP-1 antibody for further 1 h-incubation at room temperature. After washing away unbound biotinylated antibody, HRP-conjugated streptavidin (100 μl) is pipetted to the wells, followed by incubation with 100 μl of TMB One-Step Substrate Reagent in the dark room for 10 min. Finally, 50 μl of stop buffer was added into the wells and the absorbance was measured at 450 nm.

### QRT-RCR

Total RNA of cells was extracted utilizing the RNeasy Mini Kit (74,104, Qiagen, Germany) following the manufacturer’s instructions, and cDNA was acquired through reverse transcription under the assistance of PrimeScript RT reagent kit (RR036A, Takara Biotechnology, China), oligo primers (dT) and RNA (1 µg). Quantitative changes in the mRNA expression level of WISP1 was detected by Fast Start Universal SYBR Green Master (4913850001, Roche, Germany) with an ABI7900-HT-Fast device (Applied Biosystems, USA) referring to the specification of manufacturer. The sequences of the primers were listed 5’ to 3’: WISP1, (Forward (F)): AGGTATGGCAGAGGTGCAAG; (Reverse (R)): GTGTGTGTAGGCAGGGAGTG; glyceraldehyde-3-phosphate dehydrogenase (GAPDH) (F): CGCCTGGAGAAAGCTGCTA, (R): ACGACCTGGTCCTCGGTGTA. GAPDH was served as the endogenous control and the expression level was calculated by the 2^−ΔΔCT^ method [24].

### Wound healing assay

MCF7 and SK-BR-3 cells were planted in a 6-well plate at a density of 3 × 10^4^ cells/well. All cells were incubated overnight until 80–90% confluence. The next day, linear wounds were made every 0.5 cm with a pipette tip, and the cells were incubated for an additional 24 h. Images of wounds were obtained and the total number of migrated cells was counted with an Olympus BX53M microscope (×100, Japan).

### Transwell assay

The cell invasion was detected by transwell assay using Matrigel-coated transwell chambers (24-well insert; 8-μm pore size, BD Biosciences, USA). In brief, 5 × 10^4^ MCF7 and SK-BR-3 cells suspended in serum-free medium (100 μl) were added to the upper chamber, while the lower compartment was filled with 500 μl of medium containing 10% FBS. After 24 h of culturing, the cells on the basolateral side were fixed by 4% paraformaldehyde (P6148, Sigma-Aldrich, USA), dyed with 0.5% crystal violet solution (ab246820, abcam, UK) and observed under the microscope (×250).

### 5-Ethynyl-2’-deoxyuridine (EdU) assay

MCF7 and SK-BR-3 cells (1 × 10^5^ cells per well) were plated onto 12-well plates. Twenty-four hours after transfection, the cell culture medium was replaced with EdU medium (50 μm) for further 2 h of cell culture. Next, the cells were harvested and treated by 4% paraformaldehyde and 0.5% TritonX-100 (T8787, Sigma-Aldrich, USA), and stained by EdU fluorescent dye and Hoechst 33,342 from the BeyoClick EdU-594 cell proliferation detection kit (C0078S, Beyotime, China). The cell proliferation was observed under a fluorescence microscope (× 200, XSP-63XDV, Shanghai optical instrument factory, China).

## Statistical analysis

**Figure 1.**
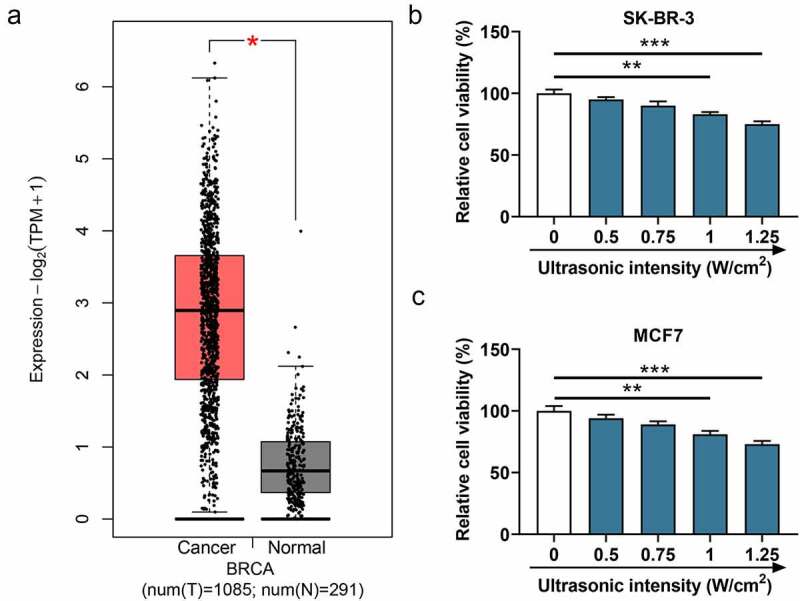
The expression of WISP1 in breast cancer tissues and the effect of ultrasonic intensity on cell viability. (a) The expression of WISP1 in breast cancer (n = 1085) and normal samples (n = 291) was analyzed by GEPIA (http://gepia.cancer-pku.cn/). (b-c) The viability of SK-BR-3 and MCF7 cells under various ultrasonic intensities (0, 0.5, 0.75, 1, 1.25 W/cm^2^) was detected by MTT assay. All experiments were repeated three times to average. The data were presented as the mean ± standard deviation (SD) of three independent experiments; *p< 0.05, **p< 0.01, ***p< 0.001. Abbreviation: GEPIA, gene expression profiling interactive analysis; WISP1, WNT1 inducible signaling pathway protein 1.

**Figure 2.**
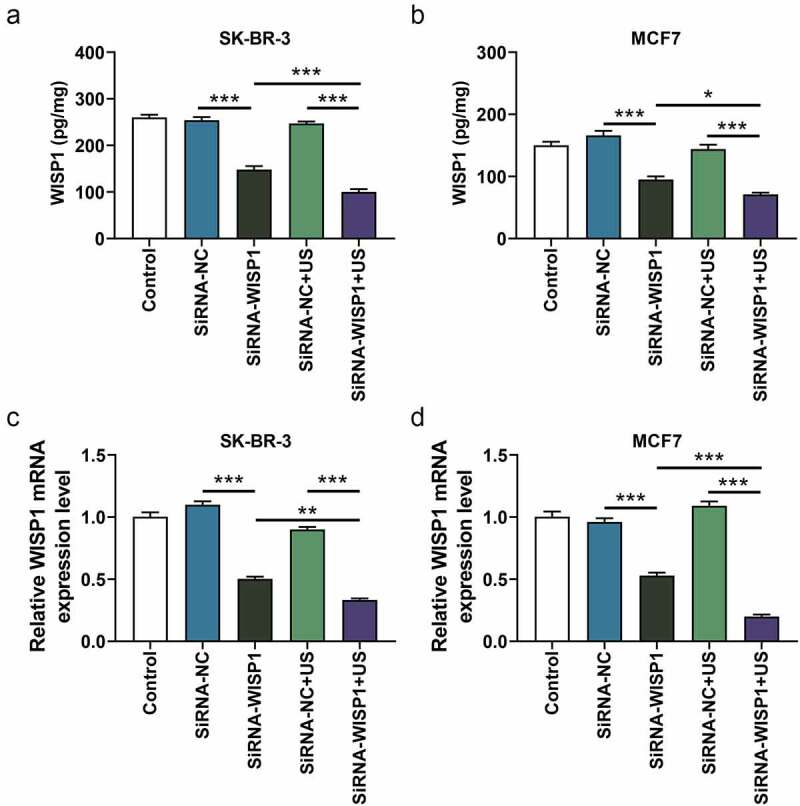
Effects of siWISP1 transfection mediated by US or Lipofectamine 6000 on WISP1 expression in breast cancer cells. (a-b) After transfection, the content of WISP1 secreted by SK-BR-3 and MCF7 cells was detected by ELISA. (c-d) The mRNA expression of WISP1 in SK-BR-3 and MCF7 cells was detected by qRT-RCR, with GAPDH serving as the internal reference. All experiments were repeated three times to average. The data were presented as the mean ± standard deviation (SD) of three independent experiments; *p< 0.05, **p< 0.01, ***p< 0.001. Abbreviation: US, ultrasonic irradiation and SonoVue microbubbles; siWISP1, WNT1 inducible signaling pathway protein 1 small interference RNA; ELISA, enzyme-linked immunosorbent assay; qRT-RCR, quantitative reverse transcription polymerase chain reaction; GAPDH, glyceraldehyde-3-phosphate dehydrogenase; NC, negative control.

**Figure 3.**
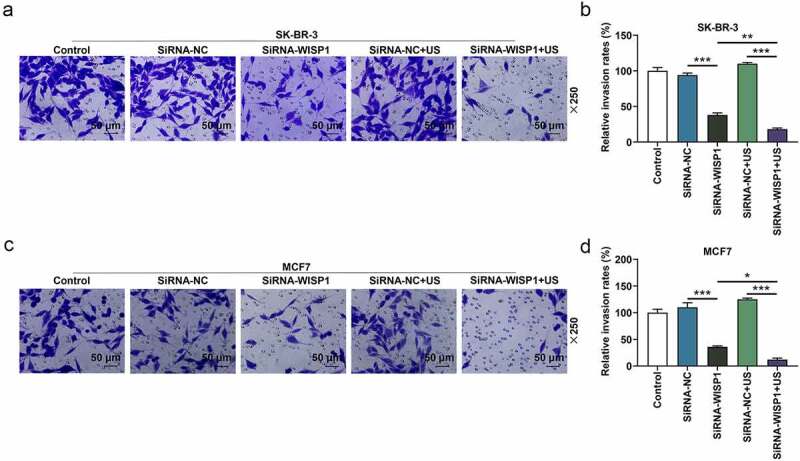
Effects of siWISP1 transfection mediated by US or Lipofectamine 6000 on invasion of breast cancer cells. (a-d) After transfection, the invasion of SK-BR-3 (a-b) and MCF7 (c-d) cells in the control, siRNA-NC, siRNA-WISP1, siRNA-NC+US and siRNA-WISP1+ US groups was detected by transwell assay (magnification ×250). Scale bar = 50 μm. All experiments were repeated three times to average. The data were presented as the mean ± standard deviation (SD) of three independent experiments; *p< 0.05, **p< 0.01, ***p< 0.001. Abbreviation: siWISP1, WNT1 inducible signaling pathway protein 1 small interference RNA; US, ultrasonic irradiation and SonoVue microbubbles; NC, negative control.

The measurement data were described as mean ± standard deviation (SD). One-way analysis of variance (ANOVA) was utilized for comparative analyses among multiple groups in Figure 1(b,c), Figure 2 and Figure 3, followed by Bonferroni method for post hoc test. All statistical analyses were implemented with Graphpad 8.0 software, and *P* < 0.05 was considered as statistically significant.

## Results

WISP1 is shown to be involved in breast cancer progression, and UTMD-mediated RNA interference is able to improve the gene transfection efficiency. We hypothesized that silencing WISP1 may suppress the biological characteristics of breast cancer cells, and that UTMD-mediated RNA interference may enhance the effects of silenced WISP1 on breast cancer cells. The goal of our study was to explore the possibility and feasibility of UTMD-delivered silenced WISP1 in the gene therapy of breast cancer. This work demonstrated that UTMD-delivered siWISP1 further attenuated invasion, migration, and proliferation of breast cancer cells in comparing with Lipofectamine 6000-transfected siWISP1.

### WISP1 expression was up-regulated in breast cancer tissues

Firstly, the breast cancer susceptibility gene (BRCA) analysis in GEPIA was utilized to determine whether WISP1 is abnormally expressed in breast cancer, and it could be concluded that the expression of WISP1 was markedly elevated in breast cancer tissues in comparison with that in normal tissues (*p*< 0.05, Figure 1a). Next, in order to enhance the effect of ultrasound and to avoid cell damage caused by ultrasound as much as possible, we examined cell viability at various ultrasound intensities (0, 0.5, 0.75, 1, 1.25 W/cm^2^) by MTT assay to screen out the optimal ultrasonic intensity. As a consequence, the viability of SK-BR-3 and MCF7 cells started to evidently decrease from the intensity of 1 W/cm^2^ (*p*< 0.01, Figure 1(b,c)), so this intensity was chosen for subsequent experiments.

***US or Lipofectamine 6000-mediated RNA interference targeting WISP1 inhibited the expression of WISP1 in breast cancer cells***

To analyze and compare the transfection efficiency of siWISP1 using US and lipofectamine 6000, the content and expression of WISP1 secreted by and in SK-BR-3 and MCF7 cells that transfected with US or Lipofectamine 6000-mediated siWISP1 were detected by ELISA and qRT-PCR, respectively. The results turned out that Lipofectamine 6000-mediated siWISP1 transfection led to the decline of WISP1 content (*p*< 0.001, Figure 2(a,b)) and expression (*p*< 0.001, Figure 2(c,d)). Moreover, compared with the Lipofectamine 6000 transfection, US-mediated siWISP1 transfection induced lower WISP1 content (*p*< 0.05, Figure 2(a,b)) and expression (*p*< 0.01, Figure 2(c,d)), indicating that US-mediated RNA interference targeting WISP1 had a more significant effect on inhibiting WISP1 content and expression in breast cancer cells than Lipofectamine 6000 transfection did.

***SiWISP1 transfection achieved by US or lipofectamine 6000 resulted in suppressed migration, invasion and proliferation of breast cancer cells***

**Figure 4.**
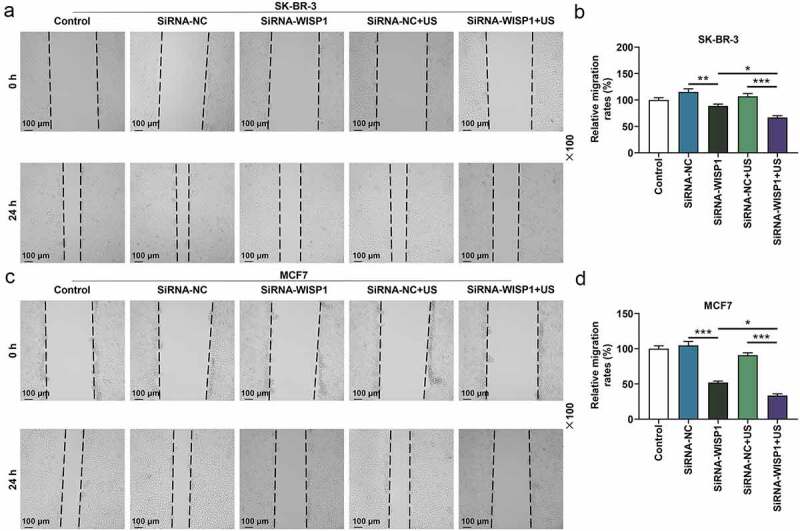
Effects of siWISP1 transfection mediated by US or Lipofectamine 6000 on migration of breast cancer cells. (a-d) Wound healing assay was used to examine the migration of SK-BR-3 (a-b) and MCF7 (c-d) cells in the control, siRNA-NC, siRNA-WISP1, siRNA-NC+US and siRNA-WISP1+ US groups (magnification ×100). Scale bar = 100 μm. All experiments were repeated three times to average. The data were presented as the mean ± standard deviation (SD) of three independent experiments; *p< 0.05, **p< 0.01, ***p< 0.001. Abbreviation: siWISP1, WNT1 inducible signaling pathway protein 1 small interference RNA; US, ultrasonic irradiation and SonoVue microbubbles; NC, negative control.

**Figure 5.**
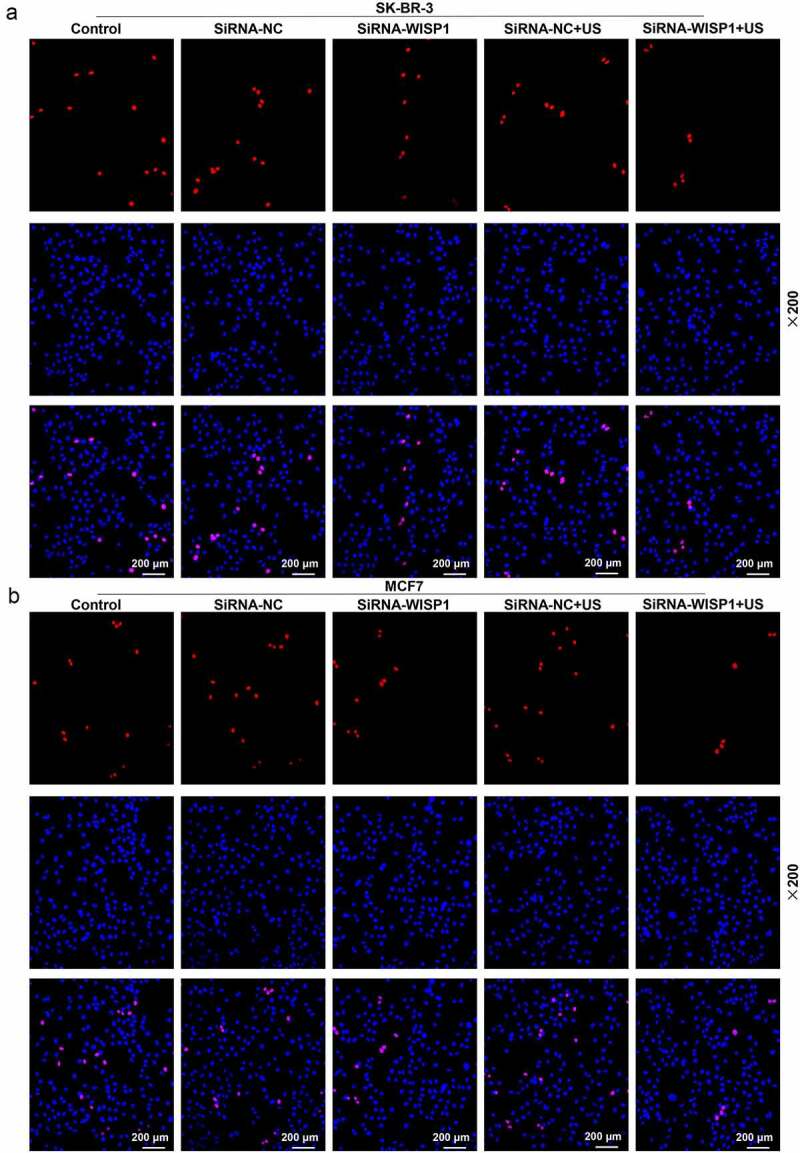
Effects of siWISP1 transfection mediated by US or Lipofectamine 6000 on breast cancer cell proliferation. (a-b) EdU assay was used to detect the cell proliferation in the control, siRNA-NC, siRNA-WISP1, siRNA-NC+US and siRNA-WISP1+ US groups (magnification × 200). Scale bar = 200 μm. Abbreviation: siWISP1, WNT1 inducible signaling pathway protein 1 small interference RNA; US, ultrasonic irradiation and SonoVue microbubbles; NC, negative control.

To further investigate the effect of US or lipofectamine 6000-mediated siWISP1 on breast cancer progression, transwell, and wound healing assays were further applied to examine the invasive and migratory characteristics of SK-BR-3 and MCF7 cells. Relative to siRNA-NC group, cell invasion (*p*< 0.001, Figure 3(a-d)) and migration abilities (*p*< 0.01, Figure 4(a-d)) were suppressed in siRNA + WISP1 group. Simultaneously, US-mediated siWISP1 transfection further inhibited the cell invasion (*p*< 0.05), Figure 3(a-d) and slowed wound closure (*p*< 0.05, Figure 4(a-d)) compared with Lipofectamine 6000-mediated transfection alone. Furthermore, EdU, as a thymine nucleoside analog, can replace thymine to penetrate into the DNA molecule being synthesized during DNA replication, thus EdU assay was used for the determination of cell proliferation. The results of EdU assay evidenced that lipofectamine 6000-mediated siWISP1 resulted in an decrease in the number of proliferating cells, and US-transfected siWISP1 had a more pronounced role on suppressing the proliferation of SK-BR-3 and MCF7 cells than lipofectamine 6000 did (Figure 5). The above results indicated that US-delivered siWISP1 has a better effect than lipofectamine 6000 on inhibiting migration, invasion, and proliferation of breast cancer cells.

## Discussion

Ultrasound microbubble targeted therapy is to use ultrasound microbubbles as the drug or gene carrier, and give a certain intensity of ultrasound irradiation in the target tissue area, resulting in microbubbles rupture to release the carried drug or gene into the tumor tissue. This is a bright method to improve the drug or gene concentration in the tumor tissue and achieve the purpose of targeted treatment toward tumor [25,26]. And there are two different signal transduction pathways in Wnt pathway: the classical pathway (mediated by Wnt-1 proteins) and the nonclassical pathway (mediated by Wnt-5a proteins) [27]. In 1998, WISP1 was identified as a downstream target gene of Wnt-1 pathway in mouse mammary epithelial cell line C57MG [28], and it is highly expressed in a variety of tumors and can regulate neuronal growth, angiogenesis, and tumorigenesis [29]. In the present study, we firstly retrieved the expression of WISP1 in breast cancer tissues by bioinformatics analysis, and uncovered its up-regulation, which was consistent with the findings of Rui Hu et al. that WISP1 was higher-expressed in breast cancer tissues than in normal breast tissues [30]. In addition, WISP1 has been proved to play an oncogenic role in breast cancer [31], and our results revealed that Lipofectamine 6000-mediated siWISP1 transfection brought about hindered invasion, migration, and proliferation of breast cancer cells. Subsequently, ultrasound microbubbles were used as carriers of siWISP1 to transfect breast cancer cells.

One of the mechanisms of ultrasound-mediated therapy is to enhance the permeability of cell membrane, which is achieved by microbubble cavitation [32]. When microbubbles are broken by inertial cavitation, the surrounding cell or tissue membrane will break due to shock wave [33,34]. Raffi Karshafian et al. illustrated the effects of ultrasonic irradiation parameters on mouse fibrosarcoma cell lines in perflutren microbubble suspension [35]. It was noticed that cell viability and membrane permeability were strongly dependent on ultrasonic irradiation parameters, namely, with the increase of peak negative pressure, pulse repetition frequency, pulse duration, and ultrasonic action time, cell membrane permeability was increased but cell viability was decreased [35]. It follows that the ultrasonic irradiation parameters affected the cell viability. In this study, viability of breast cancer cells was inhibited with increasing ultrasound intensity, coinciding with the previous research results. In addition, when the ultrasonic intensity was 1 W/cm^2^, an obvious decrease of breast cancer cell viability could be observed, so this ultrasonic intensity may be appropriate for our subsequent experiments. Nevertheless, other optimal parameters of ultrasound microbubble therapy for breast cancer should not be neglected and still need more clarification.

The key to gene transfection is how to deliver exogenous genes successfully and efficiently into the target tissues. The ideal condition is to increase the permeability of the cell membrane while the damage to surrounding tissues is reduced to a minimum, which, fortunately, can be well achieved by ultrasound microbubble transfection [36]. Zhiyi Chen et al. constructed the siRNA eukaryotic expression vector of silent human survivin gene and prepared corresponding lipid microbubbles, and *in vitro* experiments unveiled that gene-targeted microbubbles could specifically bind to high HER-2-expressing breast cancer cells and induce cancer cell apoptosis [37]. Other studies highlighted that UTMD of miR-133a could inhibit the tumor growth and improve the survival rate in breast cancer mice [38], and UTMD of Cav1.3 siRNA exerted antitumor effects on breast cancer [39]. In addition to the aforementioned literature’s illustration of the feasibility of UTMD as a gene transfection modality to treat breast cancer, another study by Hui Luo et al. evidenced that silenced PRR11 mediated by US exhibited a higher efficiency than that mediated by lipofectamine 3000 [40]. Besides, Xiaojiang Tang et al. proved that the inhibitory effect of UTMD-mediated SOCS3 on the biological behavior of breast cancer cells was better than that of liposome-mediated SOCS3 [41]. Similarly, we demonstrated that compared with the lipofectamine 6000-mediated siWISP1 transfection, the expression of WISP1 in breast cancer cells was further decreased after US-mediated delivery of siWISP1, and meanwhile the cell migration, invasion, and proliferation were further inhibited, indicating that US could enhance the transfection efficiency and promote the antitumor activity of siWISP1. Moreover, in contrast to the studies by Luo and Tang et al., our novelty lies in the first proposal that ultrasound microbubbles can serve as vehicles to deliver siWIST1 into breast cancer cells for tumor suppression.

## Conclusion

Generally, ultrasound microbubble provides an excellent platform for loading siWISP1, and siWISP1 transfection by the US, effectively inhibiting the malignant phenotype of breast cancer cells, which has a promising application prospect for breast cancer treatment. Moreover, as research continues to unravel, the involvement of WISP1 in the pathogenesis of breast cancer has been clarified, but the WISP1 regulatory network remains to be established, which thus becomes our future work direction.

## Data Availability

The analyzed data sets generated during the study are available from the corresponding author on reasonable request.
